# Spontaneous Pneumomediastinum and Cannabinoid Hyperemesis Syndrome: A Case Report and Literature Review

**DOI:** 10.7759/cureus.101979

**Published:** 2026-01-21

**Authors:** Baqir Jafry, Shawn Chillag

**Affiliations:** 1 Internal Medicine, Charleston Area Medical Center, Charleston, USA; 2 Internal Medicine, West Virginia University School of Medicine, Charleston, USA

**Keywords:** cannabis hyperemesis syndrome, macklin effect, marijuana use, nausea and vomiting, spontaneous pneumomediastinum (spm)

## Abstract

We report the case of an 18-year-old man with a six-month history of chronic marijuana use who developed spontaneous pneumomediastinum (SPM) following multiple episodes of forceful vomiting. He presented with chest pain, shortness of breath, and abdominal pain radiating to the back. A noncontrast chest CT revealed a small amount of mediastinal air without evidence of esophageal perforation, which was confirmed with a CT esophagram. He was managed conservatively with bowel rest, IV antibiotics, and supportive care. This case is one of only five to date recognizing the association between cannabinoid hyperemesis syndrome (CHS) and SPM and highlights a relatively benign condition that may not be considered in the differential diagnosis because of an uncommon underlying cause. The pathophysiology, management, and outcomes of CHS-induced SPM are discussed to place this case in context.

## Introduction

Cannabinoid hyperemesis syndrome (CHS) has been increasingly recognized in chronic cannabis users. It is characterized by cyclic vomiting, abdominal pain, and relief of symptoms with hot showers or baths [1]. Although initially regarded as benign, CHS can precipitate serious complications. One such complication is spontaneous pneumomediastinum (SPM), a condition defined by the presence of free air in the mediastinum without an apparent traumatic or iatrogenic cause [2].

This report presents a detailed account of an 18-year-old man who developed SPM following multiple episodes of vomiting, likely induced by CHS. We review the relevant pathophysiology and discuss diagnostic strategies to exclude esophageal perforation, as well as conservative management approaches described in the literature. Given the increasing prevalence of cannabis use, recognizing SPM as a potential complication of CHS is important for frontline clinicians, particularly to distinguish this generally benign condition from life-threatening causes of mediastinal air, such as esophageal perforation.

## Case presentation

An 18-year-old man with no history of significant illness or injury was transferred from an outlying facility after a chest CT revealed SPM. His symptoms included chest pain, shortness of breath, and abdominal pain radiating to the back. The day before admission to our ICU, he experienced 10-15 episodes of forceful vomiting over a 24-hour period that filled two trash cans. The vomitus consisted primarily of food contents with occasional blood streaks but no clots. He had no history of pancreatitis, NSAID use, or peptic ulcer disease. He reported a six-month history of intermittent marijuana use, with recurrent episodes of nausea and vomiting partially relieved by hot showers, and a history of occasional alcohol use, with his last alcohol intake (reported as three drinks) occurring four to five days prior.

On arrival at our facility the following day, he was hemodynamically stable and not vomiting. He was alert and oriented, in no acute distress, with clear breath sounds bilaterally and a regular cardiac rhythm. His abdomen was soft, nondistended, and nontender. No subcutaneous crepitus was appreciated, and his skin and neurologic examinations were normal.

**Figure 1:**
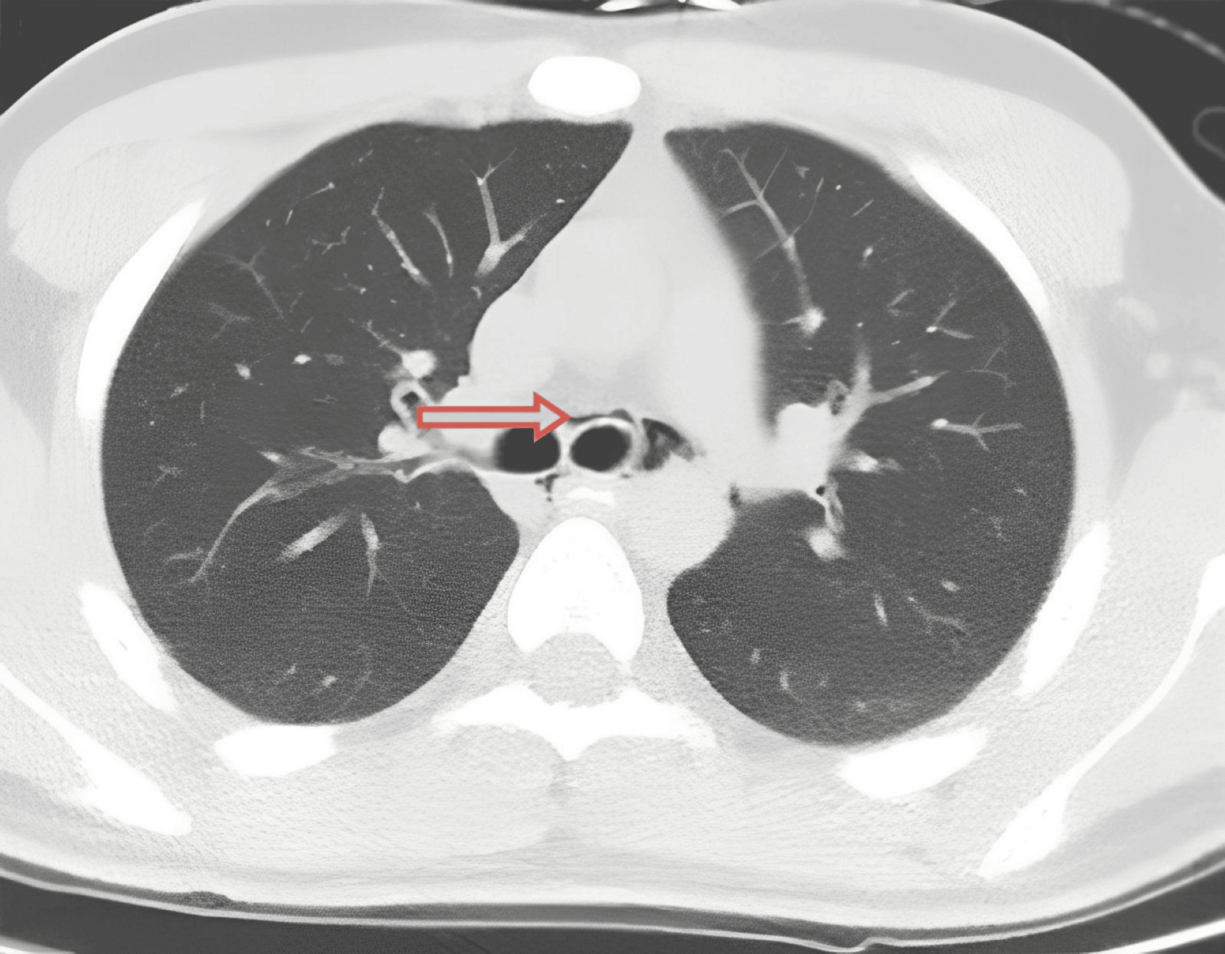
Axial CT image demonstrating pneumomediastinum A thin rim of air (arrow) is visible adjacent to the trachea (the round black airway in the center), dissecting the normally gray-white mediastinal tissue.

Initial imaging was obtained at an outside local facility (OLF) before transfer. A contrast-enhanced chest CT angiography performed there showed no evidence of pulmonary embolism and reported nonspecific pneumomediastinum in the middle mediastinum with air surrounding the mainstem bronchi. A repeat noncontrast chest CT at the admitting hospital demonstrated a small amount of pneumomediastinum with air extending into the right minor fissure (Figure 1). Laboratory studies on admission were largely unremarkable. He was admitted with a diagnosis of pneumomediastinum secondary to persistent vomiting, likely attributable to CHS.

During hospitalization, he was kept NPO with plans to advance his diet gradually and was placed on IV piperacillin-tazobactam for mediastinitis prophylaxis while esophageal perforation was being excluded. A cardiothoracic surgeon evaluated the patient and reviewed the noncontrast chest CT performed at the admitting hospital. The surgeon identified findings consistent with a Mallory-Weiss tear and possible microperforation of the esophagus. Contrast was observed within the esophagus without evidence of extravasation, effectively excluding a gross esophageal perforation.

Serial laboratory testing showed no biochemical evidence of evolving mediastinitis. At the OLF, the patient’s initial lactate level was elevated at 3.2 mmol/L, and the reflex repeat lactate normalized. A C-reactive protein level obtained at the same facility was mildly elevated (18.9 mg/L). An extended viral panel at the OLF was negative. At the OLF, the white blood cell count was elevated (20.3 × 10⁹/L); however, on admission to our hospital, the white blood cell count was normal at 9.3 × 10⁹/L and continued to trend downward to 7.8 × 10⁹/L on hospital day 2, arguing against an acute infectious process (Table 1). Hemoglobin, hematocrit, electrolyte, renal function, and glucose levels remained stable, and no metabolic abnormalities emerged. He remained afebrile and hemodynamically stable throughout hospitalization, without tachycardia, hypotension, or worsening chest pain. Physical examination revealed no new crepitus, dysphagia, tracheal tenderness, or respiratory deterioration. These stable laboratory and clinical parameters supported discontinuation of empiric piperacillin-tazobactam.

**Table 1:** Laboratory findings on the day of admission and on day 2 of the hospital stay

Parameter	Day of admission	Day 2 of stay	Reference range
White blood cell (× 10⁹/L)	9.3	7.8	4.0-11.0
Hemoglobin (g/dL)	12.8	13.8	13.5-17.5
Hematocrit (%)	37	40.5	41-53
Platelets (× 10⁹/L)	160	181	150-400
Sodium (mmol/L)	140	139	135-145
Potassium (mmol/L)	3.5	3.5	3.5-5.0
Bicarbonate (mmol/L)	26	25	22-28
Chloride (mmol/L)	108	105	98-106
Creatinine (mg/dL)	0.9	1	0.7-1.3
Blood urea nitrogen (mg/dL)	15	11	7-20
Random glucose (mg/dL)	89	96	70-140

The patient was discharged two days after admission with instructions for outpatient follow-up, including a repeat esophagram in two weeks and further evaluation of his chronic insomnia. At his follow-up visit one week later, he remained asymptomatic, had resumed a normal diet, and reported no recurrent symptoms.

## Discussion

This case recognizes the association between CHS and SPM and highlights a relatively benign condition that may not be considered in the differential diagnosis because of an uncommon underlying cause. Our patient’s clinical course mirrored that of other cases reported in the literature (Table 2) [1,3-5]. Young patients, often in their second or third decade of life, present with features that may include chest pain, dyspnea, and subcutaneous emphysema, all in the context of heavy cannabis use and repeated episodes of vomiting [1,3-5]. These cases underscore the potential for CHS to induce sufficient barotrauma to cause alveolar rupture, even in the absence of underlying lung pathology.

**Table 2:** Published cases of SPM associated with CHS CHS, cannabinoid hyperemesis syndrome; SPM, spontaneous pneumomediastinum

Case	Reference	Age/sex	Cannabis use/CHS history	Clinical presentation	Imaging findings	Management	Outcome
1	[1]	24/M	10-year heavy cannabis use; previous similar episode	Severe abdominal pain, repetitive vomiting, chest pain, neck swelling	Chest X-ray and CT: pneumomediastinum without pleural effusion; esophagram negative for leak	Topical capsaicin and IV haloperidol; 48-hour observation	Complete resolution and discharge
2	[3]	19/M	Daily cannabis use since adolescence	Recurrent vomiting (relief with hot showers), neck crepitus, chest pain	CT: pneumomediastinum; esophagram negative for esophageal rupture	Aggressive supportive therapy with IV hydration, antiemetics, sedation, continuation of hot showers	Resolution by hospital day 4; discharged in good condition
3	[4]	23/M	Daily marijuana and tobacco use	CHS symptoms with prolonged vomiting, followed by acute chest pain	Extensive pneumomediastinum with air tracking into the spinal canal (pneumorrhachis); esophagram negative for leak	Conservative management with antiemetics (promethazine), topical capsaicin	Resolution after three days; recurrence noted one year later due to cannabis relapse
4	[5]	23/F	Three-year daily marijuana use; known CHS	Intractable vomiting, retrosternal chest pain, dyspnea, subcutaneous emphysema	CT: small pneumomediastinum; esophagram negative for leak	IV fluids, antiemetics, analgesics (conservative management)	Full recovery over four days
5	Present case	18/M	Six months of chronic marijuana use; chronic insomnia	Multiple episodes of vomiting (10-15) with blood-tinged emesis, chest pain, shortness of breath, and abdominal pain radiating to the back; relief with hot showers	CT (without contrast): small pneumomediastinum with air in the right minor fissure; CT esophagram confirmed no leak; small hiatal hernia noted on CT	NPO with advancement to clear liquid diet, IV piperacillin-tazobactam, conservative management, CTS evaluation (Mallory-Weiss tear with possible microperforation), counseling on cannabis cessation	Asymptomatic at follow-up with complete resolution

This condition must be differentiated from other, more sinister causes of mediastinal air, such as esophageal perforation (Boerhaave syndrome), which carries a markedly different treatment paradigm and prognosis [6]. The collective evidence reinforces that early recognition, exclusion of esophageal perforation through appropriate imaging, and strict adherence to supportive care protocols are essential to ensure excellent short-term outcomes and prevent recurrence [1,3-5].

Chronic marijuana smoking may predispose individuals to SPM by contributing to repetitive coughing, breath-holding, and the formation of apical blebs and bullae, which are susceptible to rupture under high intrathoracic pressures [7]. However, the acute spikes in intrathoracic pressure resulting from forceful vomiting in CHS are the predominant factor leading to the development of SPM [2,8]. During severe episodes of retching, a sudden and marked increase in intrathoracic and intra-alveolar pressures can result in alveolar rupture, with air dissecting along the bronchovascular sheaths into the mediastinum, a phenomenon known as the Macklin effect [2]. Additionally, minor esophageal mucosal tears, also known as Mallory-Weiss tears, can contribute to air leakage without progressing to full-thickness perforation, as observed in Boerhaave syndrome [4,6]. Notably, despite the potential for such tears, none of the reported cases of CHS have progressed to the more catastrophic scenario of Boerhaave syndrome (Table 2).

Antiemetic therapy plays a crucial role in managing CHS-related vomiting, but was not required for our patient because he had stopped vomiting. Standard antiemetics, such as ondansetron or promethazine, are frequently ineffective, whereas alternative agents, such as haloperidol, have shown better efficacy [9]. Topical capsaicin, which mimics the relief experienced with hot showers by stimulating TRPV1 receptors, has also been successfully used as adjunct therapy [1,10].

Management of CHS-associated SPM is largely conservative. Patients are typically provided supportive care, including bed rest, administration of high-flow oxygen to expedite reabsorption of mediastinal air, and analgesia for chest pain [7]. Hospitalization is recommended for a brief period to monitor the patient and ensure that complications, such as tension pneumomediastinum, do not develop [2]. Once imaging confirms the absence of esophageal perforation, patients may be advanced from NPO status to a regular diet. In the absence of esophageal perforation, routine prophylactic antibiotics are not required, and early discontinuation is appropriate once mediastinitis has been excluded. Current evidence suggests that prophylactic antibiotics to prevent mediastinitis are unnecessary in the absence of esophageal leak [7].

## Conclusions

SPM associated with CHS, though rare, represents an important complication in chronic cannabis users. Conservative management results in excellent short-term outcomes, particularly when combined with patient education on cannabis cessation. As the prevalence of cannabis use increases, heightened clinical awareness of CHS and its complications will be crucial for prompt diagnosis and effective management.
